# Methylmercury in Industrial Harbor Sediments in Taiwan: First Observations on Its Occurrence, Distribution, and Measurement

**DOI:** 10.3390/ijerph15081765

**Published:** 2018-08-16

**Authors:** Chih-Feng Chen, Yun-Ru Ju, Guan-Ting Lin, Chiu-Wen Chen, Cheng-Di Dong

**Affiliations:** Department of Marine Environmental Engineering, National Kaohsiung University of Science and Technology, Kaohsiung 81157, Taiwan; dong3762@nkust.edu.tw (C.-F.C.); yrju@nkust.edu.tw (Y.-R.J.); plxar1001@gmail.com (G.-T.L.)

**Keywords:** mercury, methylmercury, estuary, sediments, MeHg, T-Hg, Kaohsiung

## Abstract

The distribution of methylmercury (MeHg) and total mercury (T-Hg) in sediments of the estuaries and the basin in Kaohsiung Harbor (Taiwan) is studied. MeHg in the sediment samples was determined using gas chromatography-mass spectrometry. The certified reference material of sediments with respect to the method showed the recovery efficiency between 97.4 and 103.6% which confirmed the applicability of analysis method. The T-Hg and MeHg concentrations were between 149 to 9035 μg/kg and <0.31 to 17.7 μg/kg, respectively. The T-Hg and MeHg concentrations in the estuaries of Kaohsiung Harbor were relatively high. Results suggest that Hg in this studied area was likely contributed from the catchments of the rivers. The MeHg level was <0.01 to 2.66% of the T-Hg in the sediments. A positive correlation is obtained between MeHg, T-Hg, and total organic carbon in the sediments, whereas a negative correlation is observed between pH, oxidation-reduction potential, and MeHg concentration. The results further suggest that sediment characteristics contribute mainly to the distribution of MeHg.

## 1. Introduction

The presence of mercury (Hg) and methylmercury (MeHg) in the environment is a major ecological risk due to their extreme toxicity and highly bio-accumulative properties. Some of the major sources of these materials are anthropogenic, which include agriculture, mining, incineration and municipal wastewater [1]. Due to its low solubility in receiving waters, Hg is easily adsorbed on the water-borne suspended particles. The particle-bound Hg finally accumulates in the sediments. Mercury in sediments is generally in bivalent form complexed with chlorides, sulfides or organic matter [2]. The inorganic bivalent Hg may be transformed into MeHg via the biochemical actions of local microorganisms, such as sulfate-reducing bacteria (SRB), Fe(III)-reducing bacteria, and methanogenic microorganisms [3,4,5,6,7]. MeHg can bind to proteins and pass through the biological membrane, enabling its accumulation in the food web and biomagnification, which ultimately threatens the health of wildlife and human [2,8]. Sediments are the important niche and matrix for fishes and aquatic invertebrates, hence understanding the distribution of MeHg in aquatic sediments is necessary.

Mercury methylation occurs mainly in aquatic sediments [9]. The bioavailability of inorganic bivalent Hg in the sediments and the microbial methylation and demethylation activities are the most critical factors determining the MeHg level in the sediment. Additionally, environmental conditions such as sulfur, organic matter, iron, temperature, pH, redox conditions, and salinity also play important roles in mercury methylation [10]. Under anaerobic conditions, the increase in organic matter and sulfur content are beneficial in promoting microbial activity. The bioavailability of inorganic bivalent Hg also promotes Hg methylation. However, a high sulfur content may hasten formation of Hg sulfide precipitates (e.g., HgS(s)), which in turn decreases the bioavailable Hg in the sediments and inhibits Hg methylation [2]. Therefore, environmental conditions and sediment characteristics result in variations in the distribution of MeHg in the sediments [11,12].

According to the available literature with regards to total Hg-related studies, there are few field studies on MeHg distribution in sediments. Generally, the main steps of MeHg analysis procedures for solid samples are alkaline digestion (or acid leaching), tetraethylborate derivatization for reducing the polarity of target chemical, solvent extraction by heptane, benzene, toluene, dichloromethane, or hexane, chromatographic separation by gas chromatography (GC) or high performance liquid chromatography (HPLC), and detection by mass spectrometry (MS), electron capture detector (ECD), inductively coupled plasma mass spectrometry (ICP-MS), or cold vapour atomic fluorescence spectroscopy (CVAFS) [9,12,13,14]. This study analyzes MeHg content in sediments based on the above procedures and carried out qualitative and quantitative analyses to confirm the accuracy of the analytical results.

Kaohsiung Harbor is located at the southwest coast of Taiwan. Heavy industries around this area highly depend on it due to its important role as an intercontinental container hub in logistic operations. The estuaries of Love River, Canon River, Jen-Gen River, and Salt River lie along the Kaohsiung Harbor. These four rivers are known to be contaminated with municipal sewage and industrial wastewater. Previous research indicated that the sediments in Kaohsiung Harbor were heavily polluted with Hg (0.06–6.73 mg/kg), suggesting that the main pollution sources may be these four rivers, especially the Love River and Canon River [15]. However, the distribution of the MeHg concentration in Kaohsiung Harbor and its correlation with the sediment characteristics have not been investigated. Thus, this study attempts to build a routine MeHg analysis procedure for the sediments collected from the estuary and the harbor of Kaohsiung Harbor, to understand the distribution, and pollution levels of Hg and MeHg in their sediments, and to investigate the major factors affecting the distribution of MeHg in the sediments by correlation analysis.

## 2. Materials and Methods

### 2.1. Sediment Collection and Analysis

**Figure 1:**
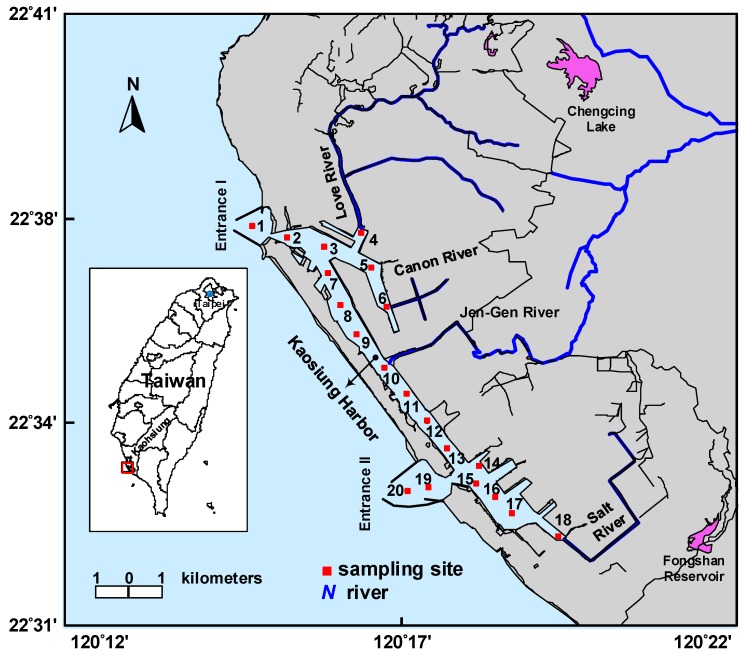
Map of the study area and location of the sampling stations.

Twenty stations were set up in in four estuaries (4: Love River, 6: Canon River, 10: Jen-Gen River, and 18: Salt River), two entrances (1: Entrance I and 19, 20: Entrance II), and navigation channels (2, 3, 5, 7, 8, and 9: the north navigation channel and 11–17: the south navigation channel) of Kaohsiung Harbor to collect the surface sediment samples (0–15 cm) using an Ekman Dredge Grab (Figure 1). The sampled sediment was put into an amber glass bottle that has been *n*-hexane washed, and held in an icebox filled with ice. When the sediment samples arrived in the laboratory, they were freeze-dried for 3 days, ground, and filtered through a 1.0 mm sieve to make them completely homogeneous.

A Coulter LS Particle Size Analyzer (Beckman Coulter, Inc., Brea, CA, USA) was used to analyze the particle size of sediment sample. Total organic carbon (TOC) was measured using the Walkley–Black method, which involves titration with ferrous ammonium sulfate of the dichromate remaining after a wet combustion of the sample with potassium dichromate [16,17]. The pH of sediment was determined by the electrode method. Sediment was mixed with water in a 1:1 ratio and continuously stirred for 5 min. Rested the mixture stand for 1 h and used the pH meter to measure the pH of the upper layer of water in the mixture [18]. ORP (oxidation-reduction potential) value in sediment was measured by redox meter immediately after sampling [19]. Briefly, a microwave digester (CEM MARS 6, Matthews, NC, USA) was used to digest 0.5 g dry sediment samples, which had been mixed with a mixture of ultra-pure acids (HNO_3_:HCl:HF = 9:3:3). The digested solution was filtered through 0.45 μm filter paper; the filtrate was diluted with ultra-pure water to a certain volume. Total mercury (T-Hg) content in sediments was determined using a cold vapor atomic absorption spectrophotometer (CVAAS) (Hitachi FHS-2 and Hitachi Z-6100, Tokyo, Japan), according to the procedure of Dong et al. [15].

### 2.2. Methylmercury Analysis in Sediment

#### 2.2.1. Chemicals and Reagents

All solvents and reagents used were of trace analysis (TA), chromatographic (HPLC) or American Chemical Society (ACS) grade. Methyl mercury chloride (MeHgCl, 95% purity), tetramethylammonium hydroxide (TMAH, 25% in water), and sodium tetraethylborate (NaBEt_4_, 99.8% purity) were all purchased from Merck (Darmstadt, Germany), while cupric acetate monohydrate (Cu(CO_2_CH_3_) 2H_2_O, 95% purity) and potassium hydroxide (KOH, 87% purity) were both obtained from Osaka Chemical Co., Ltd. (Osaka, Japan). Acetic acid (CH_3_COOH, 99.8% purity), sodium acetate anhydrous (C_2_H_3_NaO_2_, 98% purity), methanol (CH_3_OH, 99% purity), and *n*-heptane (99% purity) were acquired from Scharlau (Barcelona, Spain), Showa Chemical Industry Co., Ltd. (Tokyo, Japan), Macron Fine Chemicals™ (Center Valley, PA, USA), and ACROS (Geel, Belgium), respectively. Ultra-pure water (>18 MΩ·cm) from a Barnstead Water Purification System (Thermo Fisher Scientific, Waltham, MA, USA) was used throughout in this study.

The stock standard solution of MeHgCl (1000 mg/L) was prepared by dissolving MeHgCl in methanol. An intermediate standard solution of MeHgCl (10 mg/L) was prepared by diluting the stock standard solution with methanol. A series of calibration standard solution was prepared weekly by diluting the intermediate standard solution with ultrapure water to a range of 2.5–60 pg. All of the standard solutions were placed at 4 °C in amber glass bottles pre-washed with n-hexane and sealed with the Teflon-lined cap. The derivatization solution, i.e., 1% NaBEt_4_ solution, was prepared by dissolving 1 g NaBEt_4_ in 100 mL of 2% potassium hydroxide solution. The solution was divided into several 20 mL vials and stored at a −20 °C freezer (up to 1 month). A buffer solution (1 M) at pH 5.0 was prepared by mixing sodium acetate anhydrous and acetic acid in water. Copper solution (0.4 mM) was prepared by dissolving cupric acetate monohydrate in water.

#### 2.2.2. MeHg Analytical Procedure

Two grams (accuracy ±0.0001 g) of dry and homogenized sediment sample was put in a clean centrifuge tube (with Teflon-lined cap), and a 5 mL TMAH solution was added. Blanks were prepared following the same procedure without adding sediment sample. The standard sample used for quality control was prepared by adding the standard solution to TMAH solution. All samples were vortexed for 1 min and the mixture was subject to ultrasonic treatment using a bath sonicator (output power 200 W; frequency 40 KHz) that was filled with ice and water in ~15 °C for 15 min. The samples were neutralized by acetic acid and 1 mL of 20 mM copper solution, 5 mL of 1 M acetate buffer (pH = 5.0) and 1 mL of 1% NaBEt_4_ solution were then added. The mixture was vortexed and subjected to ultrasonic treatment. Then, 1 mL of n-heptane was added and the resulting mixture was again vortexed and subjected to ultrasonic treatment. The sample tubes were then centrifuged at 1500× *g* for 10 min. After centrifugation, the organic layer containing the target compounds was drawn with a Pasteur pipette and the extract was analyzed using gas chromatography with mass selective detection (GC-MS).

#### 2.2.3. GC-MS Instrumentation and Conditions

A GC-MS system that included an Agilent 7683B Injector, an Agilent 7890N GC, an Agilent 5975 mass selective detector (MSD) (Agilent Technologies, Santa Clara, CA, USA) and a capillary column (HP-5MS, 30 m, 0.25 mm id, and film thickness 0.25 μm) (Hewlett-Packard, Palo Alto, CA, USA) was used to separate and quantify MeHg in the sediment samples. One μL of sample was injected in splitless mode at an injection temperature of 260 °C. The transfer line and ion source temperature were at 280 °C and 230 °C, respectively. The column temperature was initially held at 45 °C for 10 min, raised to 280 °C at the rate of 20 °C/min, and finally held constant for 10 min. Detector temperature was designed 280 °C. The carrier gas of Helium was at a constant flow rate of 1 mL/min. Mass spectrum was acquired using electron ionization (EI) and selective ion monitoring (SIM) modes. The ions set to scan were 202, 217, 231, and 246 *m*/*z* for MeHg.

#### 2.2.4. Identification and Quantification of MeHg

Qualitative analysis of MeHg was conducted based on the above conditions. MeHg standard was analyzed under scan mode (scan range: 50–500 amu; scan rate: 1.6 times per sec). By comparing with the mass spectrometry database (National Institute of Standards and Technology, NIST, Gaithersburg, MA, USA), the retention time and major ions of MeHg could be defined. MeHg in the sample was qualitatively analyzed according to the retention time and the major/minor quantitative ion intensity of the authentic MeHg standards. Quantitative analysis was conducted according to the average calibration factors (CF) based on the six-point calibration curve of MeHg. The CF was calculated by dividing the peak area of major ion (217 *m*/*z*) and minor ion (246 *m*/*z*) by the concentration of MeHg. In this study, the concentration of MeHg is expressed in unit of microgram per kilogram dry-weight sediment (μg/kg dw).

### 2.3. Data Analysis

Statistical methods (i.e., mean, standard deviation, maximum, and minimum) were used in data analysis. The concentration distribution diagram of MeHg and T-Hg were sketched with Surfer^®^ Version 8.0 software (Golden Software Inc., Golden, CO, USA, 2002). The correlation among all the sediment characteristics (e.g., grain size, pH, ORP, and TOC) and Hg concentrations (MeHg and T-Hg) determined by the Pearson correlation coefficient using the statistics software SPSS 12.0.

## 3. Results and Discussion

### 3.1. Quality Assurance and Quality Control (QAQC) for Methylmercury Analysis

The six-point calibration curve (2.5 to 60 pg), procedural blank, check standard, and sample duplicates were carried out for each set of samples. The calibration factor based on the six-point calibration curve for MeHg showed acceptable relative standard deviation (RSD) values (4.1%). All of procedural blank values were lower than the detection limit. The recovery of MeHg in check standards ranged from 82.3 to 101.1% (n = 6), and the relative percent difference of sample duplicates ranged from 4.0 to 8.6% (n = 4) for the analyses of MeHg (Table 1). The detection limit of the analytical procedure was estimated from three times standard deviation from repeated (n = 7) analysis of MeHg (2.5 pg), and the amount of sample extracted. The detection limit of MeHg was 0.31 μg/kg dw (Table 1). The reference material ERM-CC580 (estuarine sediment) from Institute for Reference Materials and Measurements (IRMM), European Commission—Directorate General Joint Research Centre (Geel, Belgium) was used. Table 2 shows certified and measured concentrations. There is a good agreement between certified and measured results and the recovery was between 97.4% and 103.6% for MeHg.

### 3.2. GC-MS Separation and Identification

**Figure 2:**
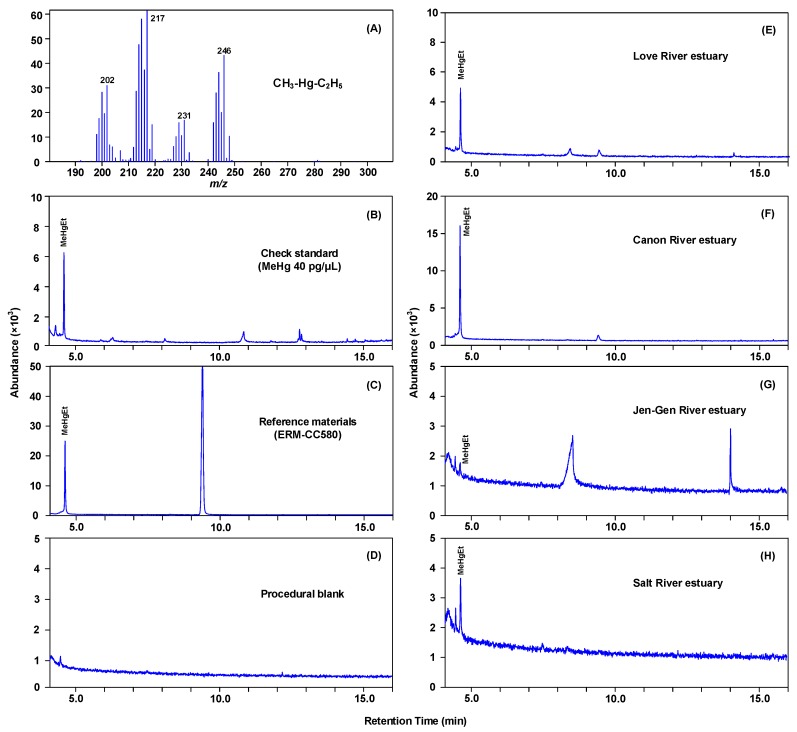
(**A**) The electron impact mass spectra for GCMS analysis of MeHgCl standard solution derivatizing with NaBEt4; chromatogram of the selected major ion 217 *m*/*z* for (**B**) the check standard, (**C**) reference material (ERM-CC580), (**D**) procedural blank, and four estuaries of (**E**) Love River, (**F**) Canon River, (**G**) Jen-Gen River, and (**H**) Salt River.

Prior to analyzing the sediment samples, the efficiency of GC-MS for analyzing MeHg concentration was tested with a MeHgCl standard solution. The MeHgCl standard solution was derivatized by NaBEt_4_, extracted by *n*-heptane, followed by GC-MS analysis in scan mode. The retention time of the MeHgEt, the derivative of MeHgCl standard, was 4.612 min. Figure 2A shows the mass spectra of MeHgEt, the derivative of MeHgCl. The major ion was 217 *m*/*z*, and the minor ions included 202, 231, and 246 *m*/*z*. The identity of MeHg was confirmed by the retention time and abundance of quantification ions in the authentic MeHg standards. The separation and quantitation of MeHg in the sediment samples were achieved using the same GC-MS conditions as the standards. The MeHg was quantified using the calibration factor based on the six-point calibration curve. Figure 2B–H shows the sample chromatogram of the selected major ion 217 *m*/*z* for the check standard, reference material (ERM-CC580), procedural blank, and four estuaries. The peak of MeHg for all samples was clearly separated without noise interfering at the retention time of 4.61 min.

### 3.3. Sediment Characteristics

**Figure 3:**
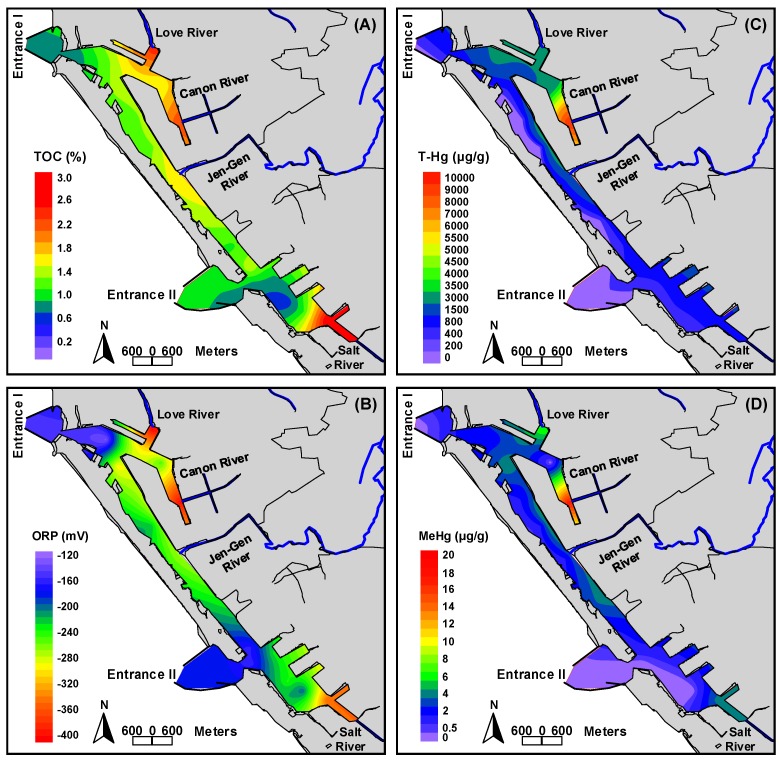
The distributions of (**A**) TOC content, (**B**) ORP, (**C**) T-Hg, and (**D**) MeHg concentrations in the sediments of Kaohsiung Harbor.

Table 3 lists the distributions of sediment characteristics of the 20 stations, including grain size, TOC, ORP, and pH. The dominate composition of grain size in sediments collected from the studied area was the fine particle (clay and silt, <63 μm), accounting for 55.1–90.4%, except for stations 1, 2, 6, 7, 11, and 15 which were dominated by sand (>63 μm) with about 57.8–100%. The higher TOC content was found in the sediments of estuaries with 1.72–2.50%, whereas the relatively lower one (0.71–1.63%) was found in the remaining stations, including channels and entrances (Figure 3A). On the contrary to the distribution of TOC, pH values in the sediments of estuary stations (pH: 7.31–7.52) were relatively lower than those in the remaining stations (pH: 7.31–7.81) (Table 3).

Negative ORP values were measured in all sampling stations (−129–−392 mV) especially the estuary stations with high TOC content (ORP: −254–−392 mV) (Figure 3B). This indicates that the sediments of Kaohsiung Harbor are in anaerobic state, which can be attributed to the microbial mediated oxidation of the organic matter consuming oxygen in the sediments. The lower pH value and higher TOC content were found in the sediment of estuary stations because these stations receive the inputs from freshwater of rivers and terrigenous organic matter. The higher TOC content was associated with the reducing state of the sediment.

### 3.4. Distribution of Total Mercury and Methylmercury in Sediments

The T-Hg concentrations ranged from 149 to 9035 μg/kg with mean ± SD of 1072 ± 1957 μg/kg in all 20 sampling stations. The higher T-Hg concentrations were found at the area around station 3 to station 6 (1699–9035 μg/kg), especially for station 6 which presented a high T-Hg concentration of 9035 μg/kg; whereas the remaining stations showed T-Hg concentrations lower than 1000 μg/kg (149–896 μg/kg) (Table 3). The T-Hg concentration measured in this study was comparable with the effect range low (ERL: 150 μg/kg) and effect range median (ERM: 710 μg/kg) published by the US National Oceanic and Atmospheric Administration [20]. All of the detected T-Hg concentrations in the sediment exceeded the ERL value of 150 μg/kg. The T-Hg concentrations at stations 3, 4 (Love River estuary), 5, 6 (Canon River estuary), and 10 had also exceeded the ERM value of 710 μg/kg, indicating that the levels of T-Hg in the sediments may adversely impact the benthic organisms. Figure 3C illustrated the distribution of T-Hg concentrations in the sediments of Kaohsiung Harbor. It can be found that the major hot pollution zone was around the Canon River estuary (station 6). The T-Hg concentration progressively decreased toward the channel and the entrances. Result of the distribution of T-Hg clearly demonstrated that Hg accumulated in the sediments along the harbor area came from freshwater inputs. The main pollution sources may be municipal sewage and industrial wastewater discharges to the river upstream or along the river (e.g., metal processing, plastics, paint and dye, chemical manufacturing, electronics, motor vehicle plating and finishing, paper and board mills, and foundries) [15,21].

MeHg concentration was detected in 14 of the 20 sediment samples collected from the studied area with the range between 0.61 and 17.7 μg/kg. The highest MeHg concentrations were observed in station 4 (Love River, 6.81 μg/kg) and 6 (Canon River, 17.7 μg/kg) (Table 3). The spatial distribution pattern of MeHg in the sediment was similar to that of T-Hg, TOC content, and ORP (Figure 3). Previous studies had indicated that MeHg concentration in the sediment was associated with T-Hg because MeHg in the sediments mainly was transferred by the bioavailable Hg via the biochemical effects [12,22]. Therefore, the sediments with the higher T-Hg concentration generally exhibited relatively higher MeHg concentration [12,23,24]. Moreover, the estuary sediments with high TOC content and the highly anaerobic state could promote the activity of microorganism, which favors MeHg formation resulting in relatively higher MeHg concentration in the above environment [2]. In this study, the sediments in the estuary stations had relatively higher level of T-Hg, TOC content, and redox state, especially for stations 4 (Love River estuary), 6 (Canon River estuary), and 18 (Salt River estuary); these stations exhibited the relatively higher MeHg concentration. Results showed the ratio of MeHg concentration to T-Hg (MeHg ratio) ranged from <0.01 to 2.66, with the mean ± SD of 0.42 ± 0.60. The MeHg ratio at stations 7–9, 11–12, and 18 (0.58–2.66) was relatively higher than that at other stations (0.01–0.32), indicating that the sediment at those stations contained a higher portion of bioavailable inorganic Hg, thus resulting in a higher portion of MeHg. Overall, the MeHg ratio of the sediment in Kaohsiung Harbor was similar to that in general estuary and marine about <1.5% [9,25,26].

The concentrations of T-Hg and MeHg obtained in this study were compared with those available in the literature to understand the level of Hg pollution in Kaohsiung Harbor. As shown by the data listed in Table 4, the concentrations of T-Hg and MeHg in the sediments of Kaohsiung Harbor were slightly lower than that of the Gulf of Trieste and the Gulf of Taranto (Italy), Nerbion-Ibaizabal Estuary (Spain), and Minamata Bay (Japan), but were relatively higher than other regions of the world. Additionally, MeHg ratio estimated in this studied was consistent with most of the other regions, lower than 1% (Table 4). Theoretically, the higher T-Hg concentration of sediments in Kaohsiung Harbor should provide the higher probability to produce MeHg but results were exactly the opposite. The phenomenon has been mentioned that high concentration of inorganic Hg may inhibit the methylation activity of microorganism and promote the demethylation, resulting in the MeHg ratio in sediments being limited in a certain range of 1% [26].

### 3.5. Correlation of Sediment Characteristics and Total Mercury and Methylmercury

The sediment characteristics (such as grain size, TOC, pH, and ORP) play an important role in the distributions of T-Hg and MeHg in the sediments, the correlation between the two is proposed above [12,15,40,41]. Table 5 lists the Pearson correlation matrix for the sediment characteristics, T-Hg, MeHg, and MeHg ratio. The T-Hg concentration in the sediments has no relation to grain size (*p* > 0.05, r = −0.329–0.274) but was significantly and positively correlated to the TOC content (*p* < 0.05, r = 0.500), indicating that the mechanism for sediment adsorption of Hg was dominantly determined by the chemical adsorption, not the physical surface adsorption [42]. A significantly negative relation was exhibited for the T-Hg concentration with pH (*p* < 0.05, r = −0.510) and ORP (*p* < 0.05, r = −0.553) in the sediments. It could be inferred that Hg and organic pollution in the sediments of Kaohsiung Harbor were mainly contributed from the riverine freshwater, accumulated in the estuary, and gradually spread to the harbor area. This may be the reason for the negative correlation between the T-Hg concentration in the sediment and the pH and ORP. The MeHg concentration in the sediments was negatively related with TOC (*p* < 0.01, r = 0.653), pH (*p* < 0.05, r = −0.511), and ORP (*p* < 0.01, r = −0.711). TOC content in the sediment is one of the important factors affecting the Hg methylation process [2]. The organic matter-rich sediment generally contains relatively higher MeHg level, because the dissolved organic matters (i.e., electron donor) enhance the activity of microorganism that promotes the Hg methylation process [2,43].

In addition, the sediments with high TOC content generally had relatively lower pH and ORP value due to the microbial oxidative decomposition. The lower pH may increase the solubility and mobility of Hg and the sediments under anaerobic state (i.e., low ORP), which is favorable to microbial Hg methylation [2,44]. Therefore, TOC content in the sediments both directly and indirectly influences the formation of MeHg. A significantly linear relationship between T-Hg and MeHg concentrations (*p* < 0.01, r = 0.889) suggests that the sediments with the high T-Hg content could provide the relatively more inorganic Hg(II) (i.e., bioavailable Hg) for microbial methylation that results in increasing MeHg concentration with increasing T-Hg [12,23,24].

No significant correlation was found between MeHg ratio and all other measured parameters, indicating that it could have the other critical factor affecting the MeHg ratio. The MeHg ratio is mainly determined by the activities of microorganism relatively for methylation and demethylation and the proportion of bioavailable inorganic Hg in the sediments. However, determining the net rate of methylation and demethylation processes includes a network of biogeochemical reactions and environmental conditions [26,45]. The main factors affecting the Hg methylation are numerous and complex, including microorganisms, sulfide, organic matter, iron, selenium, pH, temperature, drying and wetting cycles, the age of Hg, redox stations, and salinity. Therefore, the main factors to determine the MeHg ratio may be an unmeasured item from the above factors or a combination of all the above factors [2,26].

## 4. Conclusions

Analysis for T-Hg and MeHg in the sediments of harbor and estuary in Kaohsiung Harbor (Taiwan) was carried out. The distributions, levels of T-Hg and MeHg, and the correlation analysis with sediments characteristics were also evaluated. The T-Hg and MeHg concentrations in the sediments were between 149 and 9035 μg/kg as well as <0.31 and 17.7 μg/kg, respectively. Relatively higher concentrations of T-Hg and MeHg were found in the sediments of the estuaries in Kaohsiung Harbor. The results suggested that Hg in the study area likely originated from the river catchments. The MeHg level was <0.01 to 2.66% of the T-Hg concentration in the sediments. There is a positive correlation between MeHg and T-Hg concentrations and TOC content, but a negative correlation was observed between pH and ORP values with the MeHg concentrations in the sediments of the studied area. This study can be useful for the MeHg monitoring in the sediments of the harbor and for the design of future strategies in environmental protection of the harbor environments, with a special focus on the area at the estuary.

## Figures and Tables

**Table 1:** Calibration factor, detection limits, recoveries of check standards, and relative percent differences of sample duplicates for MeHg analysis.

Calibration Factor (CF) (n = 6)		Detection Limits (μg/kg dw)	Blank Analysis (n = 6) (μg/kg dw)	Check Analysis (n = 6) R ^1^ (%)	Duplication Analysis (n = 4) RPD ^1^ (%)
Average ± SD ^1^	RSD ^1^ (%)				
2513 ± 103	4.1	0.31	<DL	91.8 ± 6.9	6.2 ± 2.4

^1^ SD: standard deviation; RSD: Relative standard deviation; R: Recoveries; RPD: Relative percent differences.

**Table 2:** Analysis of methylmercury from reference materials ERM-CC580 (estuarine sediment).

Compounds	Measured Value (μg/kg dw)				Certified Value (mean ± SD)
	#1	#2	#3	Mean ± SD ^1^	
MeHg	77.2	72.6	73.8	74.5 ± 2.4	75.5 ± 4.8

^1^ SD: standard deviation.

**Table 3:** The basic characteristics, total mercury (T-Hg), and methylmercury (MeHg) in sediment samples collected from the estuarine and channel of Kaohsiung Harbor.

Site	Clay	Silt	Sand	TOC	pH	ORP	T-Hg	MeHg	MeHg Ratio ^1^
	(%)	(%)	(%)	(%)		(mV)	(μg/kg)	(μg/kg)	(%)
1	<0.01	<0.01	100.0	0.92	7.77	−146	401	0.61	0.14
2	4.3	37.9	57.8	0.86	7.55	−143	702	2.49	0.33
3	7.5	82.9	9.6	1.33	7.31	−129	1699	2.55	0.14
4	4.2	51.7	44.1	2.50	7.32	−392	1988	6.81	0.32
5	6.2	72.9	20.9	1.63	7.40	−245	1944	<0.31	0.01
6	2.3	16.2	81.5	2.39	7.31	−385	9035	17.67	0.18
7	2.2	14.3	83.5	1.53	7.50	−286	472	4.94	0.97
8	7.6	60.3	32.1	1.31	7.61	−252	298	2.20	0.69
9	7.7	79.0	13.3	1.36	7.45	−215	297	1.99	0.62
10	7.1	71.2	21.8	1.72	7.43	−254	896	2.02	0.21
11	4.0	35.8	60.2	1.47	7.45	−222	149	4.26	2.66
12	5.4	49.8	44.8	1.17	7.78	−199	374	2.33	0.58
13	5.5	52.1	42.4	1.52	7.74	−158	646	1.75	0.25
14	8.2	69.2	22.6	1.02	7.81	−220	521	1.58	0.28
15	4.9	33.5	61.6	0.91	7.70	−157	223	<0.31	0.06
16	7.4	66.9	25.8	0.71	7.58	−231	447	<0.31	0.03
17	7.6	69.3	23.1	1.17	7.49	−193	449	<0.31	0.03
18	6.6	68.1	25.3	2.84	7.52	−338	524	4.19	0.75
19	6.4	48.7	45.0	1.01	7.65	−179	199	<0.31	0.07
20	6.9	61.2	31.8	1.07	7.64	−174	173	<0.31	0.08

^1^ MeHg ratio = T-Hg/MeHg as Hg; using 1/2 DL as the calculating concentration when the concentration of MeHg was below to DL.

**Table 4:** Total mercury (T-Hg), methylmercury (MeHg), and the ratio of MeHg to T-Hg (MeHg ratio) in the sediments of the different regions of the world.

Location	T-Hg (μg/kg)	MeHg (μg/kg)	MeHg Ratio (%)	Reference
Biscayne Bay, USA	17	0.026	0.15	[11]
Tampa Bay, USA	8.3	0.049	0.59	[11]
Charlotte Harbor, USA	29	0.074	0.26	[11]
Florida Bay, USA	12	0.082	0.68	[11]
Pine Island Sound, USA	6.3	0.055	0.87	[11]
Hudson river, USA	1000	1.3	0.13	[27]
San Francisco Bay, USA	200	0.4	0.2	[28]
Narragansett Bay, USA	555	1.852	0.42	[29]
Gulf of Trieste, Italy	5240	16.9	0.32	[30]
Gulf of Taranto, Italy	2770	10.8	0.39	[31]
Vistula Mouth, Poland	71	0.354	0.50	[23]
Odra mouth, Poland	9	0.075	0.83	[23]
Gulf of Gdansk, Poland	164	0.645	0.39	[32]
Anadyr Estuary, Russia	339	0.254	0.22	[32]
Mediterranean Basin	110	1.33	1.21	[33]
Nerbion-Ibaizabal Estuary, Spain	2005	46.8	2.33	[34]
Taheri Port, Iran	34	0.2	0.69	[35]
Dayyer Port, Iran	26	0.2	0.65	[35]
Persian Gulf, Iran	20	0.14	0.71	[36]
Dora, Lebanon	100–650	0.07–0.5	0.03–0.35	[37]
Minamata Bay, Japan	2960	1.74	0.06	[24]
Isahaya Bay, Japan	66	0.11	0.16	[24]
Off Kuala Terengganu, Malaysia	61	0.038	0.11	[32]
Victoria Harbor, Hong Kong	247	<0.1–1.5	0.14	[38]
East China Sea, China	37	2.7	8.8	[39]
Fujian and Guangdong coasts, China	31.5	0.096	0.30	[22]
Kaohsiung Harbor, Taiwan	1072	2.86	0.42	This study

**Table 5:** Pearson correlation matrix for the characteristics, T-Hg, MeHg, and MeHg ratio in the sediments of Kaohsiung Harbor.

Item	Clay	Silt	Sand	TOC	pH	ORP	T-Hg	MeHg
Silt	0.939 ^a^							
Sand	−0.949 ^a^	−1.000 ^a^						
TOC	−0.140	−0.032	0.017					
pH	0.000	−0.198	0.181	−0.571 ^a^				
ORP	0.159	0.077	−0.085	−0.828 ^a^	0.515 ^b^			
T-Hg	−0.329	−0.267	0.274	0.500 ^b^	−0.510 ^b^	−0.553 ^b^		
MeHg	−0.441	−0.392	0.398	0.653 ^a^	−0.511 ^b^	−0.711 ^a^	0.889 ^a^	
MeHg ratio	−0.199	−0.199	0.220	0.177	−0.129	−0.154	−0.171	0.172

^a^ Correlation is significant at the 0.01 level (2-tailed). ^b^ Correlation is significant at the 0.05 level (2-tailed).
